# Seed‐Based Rehabilitation of *Phytophthora cinnamomi*‐Infested Forest Sites

**DOI:** 10.1002/ece3.70900

**Published:** 2025-02-24

**Authors:** Himbutugoda S. Harshani, Todd E. Erickson, Jen McComb, Treena Burgess, Giles Hardy

**Affiliations:** ^1^ Phytophthora Science and Management, Harry Butler Institute Murdoch University Murdoch Western Australia Australia; ^2^ Centre for Engineering Innovation: Agriculture and Ecological Restoration, School of Agriculture and Environment University of Western Australia Crawly Western Australia Australia; ^3^ Kings Park Science, Department of Biodiversity Conservation and Attractions Kings Park Western Australia Australia; ^4^ ArborCarbon, ROTA Compound Murdoch University Murdoch Western Australia Australia

**Keywords:** extruded pellets, jarrah forest, *Phytophthora*‐dieback, resistant species, revegetation, seed enhancement

## Abstract

The plant pathogen *Phytophthora cinnamomi* has significantly damaged the floristic diversity and community structure of the jarrah ( *Eucalyptus marginata* ) forest in Western Australia. Complete eradication of the pathogen from infested sites is not possible. This study assessed the feasibility of rehabilitating *P. cinnamomi*‐infested forest sites with native resistant species using various methods of seed deployment. Precision burial of seeds at 5 mm was used as a control, mimicking optimum recruitment depths for many native species and compared against the use of extruded pellets (hereafter ‘pellets’) as an alternative method of precision seed placement. Eighteen rehabilitation plots were set up in three *P. cinnamomi*‐infested reserves using six species. For *Acacia acuminata* , *A. saligna* , *Calothamnus sanguineus* and *Melaleuca seriata* , there were three treatments: precision buried (non‐pelleted) seeds, pellets and pellets with an additive (i.e., a rhizobium bacterium for the *Acacia* spp. and ectomycorrhizal fungus spores for *C. sanguineus* and *M. seriata* ). *Banksia sessilis* and *Hakea laurina* had only two treatments: precision buried (non‐pelleted) seeds and pellets. Seedlings of all six species emerged successfully in *P. cinnamomi*‐infested sites, and the numbers ranged between 23% and 88%. The survival of emerged seedlings after 9 months ranged between 16% and 84%, except *M. seriata* , which emerged at 59% but failed to survive. In most species, except *A. acuminata* , seedling emergence and survival from pellets were similar and within an acceptable seedling establishment range when compared to non‐pelleted seeds. Pelletised seed with the addition of beneficial microbes did not improve seedling survival or shoot growth in the diseased areas of the jarrah forest. Overall, the results suggest that seedlings of resistant native species can successfully establish in *P. cinnamomi*‐infested sites and pelletised seeds can be used as a viable method for precision planting.

## Graphical abstract

This article found that seedlings of resistant native species can successfully establish in *Phytophthora cinnamomi*‐infested sites and pelletised seeds can be used as a viable method for precision planting in dieback rehabilitation projects.

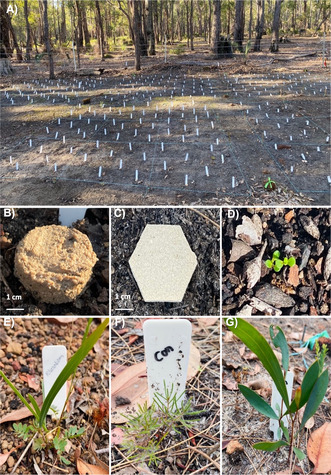

## 1 Introduction

Plant pathogens are a major cause of tree decline in forest ecosystems across the globe (Rizzo and Garbelotto 2003). *Phytophthora cinnamomi* Rands is an invasive soil‐borne plant root pathogen that causes deaths in forest ecosystems across Europe (Vettraino et al. 2005; Scanu et al. 2013), North America (Balci, Bienapfl, and Lamour 2013), Africa (Brasier 1999) and Australia (Hardham 2005). In Australia, many native species, in a wide array of plant communities in all states, are susceptible to the pathogen (Cahill et al. 2008) and it is listed as a key threat to Australian biodiversity (Department of Environment 2014).

In Western Australia, more than a million hectares have been impacted by the pathogen (Department of Environment 2014). One severely impacted ecosystem is the jarrah ( *Eucalyptus marginata* Sm.) forest, a biodiversity‐rich wet sclerophyll forest (McCaw, Robinson, and Williams 2011; Macintyre and Mucina 2021). *Eucalyptus marginata* and *Corymbia calophylla* (Lindl.) K.D.Hill & L.A.S.Johnson are the two most dominant overstorey species, and the forest has a highly diverse mid‐ and understorey vegetation (Koch 2007). Approximately 20% of the forest has been infested by the pathogen (Jung, Colquhoun, and Hardy 2013). A high proportion of the mid‐ and understorey species are from the Asphodelaceae (syn. Xanthorrhoeaceae), Dilleniaceae, Epacridaceae, Fabaceae and Proteaceae, which are highly susceptible to the pathogen, and therefore, the impact to the understorey vegetation is severe (Shearer and Tippett 1989; Shearer and Dillon 1995).

Reduced species richness and vegetation cover, local extinction of species and increased weeds in pathogen‐infested areas have changed the structure and composition of the forest (McDougall, Hobbs, and Hardy 2002; Shearer et al. 2007; Harshani, Tsakalos, et al. 2023). The changes in the floristic diversity and community structure have reduced the availability of habitats and resources for dependent fauna and flora (Garkaklis et al. 2004). Pathogen‐caused plant deaths alter litter accumulation and decomposition, nutrient cycling, water cycling, the overall ecosystem functioning and the forest's health (Shearer et al. 2009).

It is possible to eliminate the pathogen from small, isolated areas of native vegetation and mine sites (Dunstan et al. 2010, 2020). However, complete eradication of *P. cinnamomi* from large forest areas is impossible. Therefore, current management measures focus on minimising the disease spread to non‐infested areas or mitigating the impact in diseased areas (O'Gara et al. 2005).

The re‐establishment of diverse understorey vegetation in diseased forest sites is essential to restore healthy ecosystem functioning, but current management practices do not address this. There have been few attempts to establish plants in *Phytophthora*‐infested sites, and they have focused only on overstorey species. Fagg (1987) conducted two trials in *Phytophthora*‐infested sites in eucalypt forests of East Gippsland in Victoria, sowing seeds of six eucalypt species, four species known to be tolerant to the pathogen and two susceptible. Satisfactory seedling establishment was observed for both trials after 7 and 4.7 years, respectively. Susceptible and resistant clones of *E. marginata* were planted in bauxite mine sites in the jarrah forest and inoculated with *P. cinnamomi*. After 13 years, there were 40%–100% deaths of susceptible clones and 0%–30% of resistant ones (Stukely et al. 2007). However, these studies were conducted in either mined landscapes from which all vegetation had been removed (i.e., bauxite mine study) or considerable site preparation had occurred that include burning and ploughing (i.e., East Gippsland study). While significant research has been conducted on the impact of *P. cinnamomi* in the jarrah forest, recent investigations are limited. Moreover, there are no reports on attempts to improve species diversity in the understorey of diseased jarrah forest sites with remnant vegetation, using resistant species. Similarly, apart from Harshani, McComb, et al. (2023), there has been little recent research on the susceptibility or resistance of native plant species at the early establishment stage.

For rehabilitation of large areas, direct seeding is preferred over planting seedlings due to the higher cost required to grow seedlings under ex situ nursery conditions, and plant them in situ (Pérez et al. 2019). Seeding is typically done by broadcasting seeds either manually or mechanically, or by direct sowing them using seeding machines designed for precision placement belowground so that seeds are placed at depths (e.g., 5–10 mm) known to maximise recruitment potential (Ott, Cox, and Shaw 2017; Masarei et al. 2019). Broadcasted seeds left on the soil surface often get predated or removed from the targeted site by wind or rain (DeFalco et al. 2012; Pearson et al. 2019), resulting in very low seedling establishment rates (Ott, Cox, and Shaw 2017). Consequently, there has been an increased focus on using precision seeding machines (Erickson et al. 2019; Masarei et al. 2021).

Using precision seeding machinery in *Phytophthora*‐infested sites is not feasible for several reasons. Large overstorey trees and some resistant species in these sites make it impractical to access and operate such machinery. Moreover, transporting machinery from infested sites through healthy areas increases the risk of spreading the disease. While precision seeding by hand is an option, it is neither practical nor cost‐effective for large‐scale rehabilitation projects. Therefore, extruded seed pelleting was evaluated as one potential method for facilitating precision seed distribution. The pellets can aid the distribution of seeds of various sizes and shapes, provide a microsite for seed germination where seed‐soil contact is maximised pre‐sowing and enhance seedling emergence and establishment (Madsen et al. 2016; Erickson et al. 2021). It also allows the provision of beneficial additives to the seeds, including germination stimulants, beneficial soil microbes, predation deterrents and fungicides (Erickson et al. 2021; Dadzie et al. 2022).

This study investigated seed‐based rehabilitation of *Phytophthora*‐infested sites in the jarrah forest using six understorey native species, *Acacia acuminata* Benth., *A. saligna* Labill., *Banksia sessilis* (Knight) A.R.Mast & K.R.Thiele, *Calothamnus sanguineus* Labill., *Hakea laurina* R.Br. and *Melaleuca seriata* Lindl. Rehabilitation plots were established in *Phytophthora*‐infested sites to (1) evaluate whether the seedlings can emerge and survive in forest sites where the pathogen is present, (2) evaluate whether extruded seed pelleting can facilitate seed distribution and achieve seedling emergence and survival comparable to that of precision seed burial and (3) if the addition of rhizobium bacterium or ectomycorrhizal fungus in extruded pellets benefits seedling survival and growth.

## 2 Materials and Methods

### 2.1 Study Sites

The study was conducted in three bushland reserves in the Northern Jarrah Forest from July 2022 to April 2023 (Table 1). The reserves are in the Darling Escarpment, all within 15 km of each other, and approximately 35 km east of Perth, Western Australia. The reserves are severely impacted by *P. cinnamomi* (Harshani, Tsakalos, et al. 2023), and the presence of the pathogen was confirmed by a *Phytophthora* dieback assessment conducted in these reserves (Terratree Pty Ltd. 2020). *Phytophthora*‐infested sites in Falls Road reserve had the highest level of remnant vegetation, while the remaining vegetation in the Black Cockatoo and Quail Street *Phytophthora*‐infested sites was sparse. Each reserve had been control burnt in 2012. The study area has a Mediterranean climate (Bradshaw 2015), and the highest rainfall during the study was between July and September 2022 (Appendix S1: Figure S1). The mean minimum and maximum temperatures during the study were 7.1°C and 31.5°C, respectively (Appendix S1; Australian Bureau of Meteorology 2023).

**TABLE 1:** Description of the reserves selected for the rehabilitation experiment.

Reserve	Location	Vegetation type^a^	Soil type^b^	Total area (ha)^c^	*Phytophthora* status^c^		
					Infested (%)	Non‐infested (%)	Uninterpretable (%)
Black Cockatoo	−31.895° S 116.179° E	Yarragil	Murray 2 Phase	13.01	69.9	30.1	0
Falls Road	−31.877° S 116.124° E	Dwellingup	Dwellingup 2 Phase	19.68	61.5	29.9	8.6
Quail Street	−31.829° S 116.220° E	Yarragil	Dwellingup 2 Phase	105.2	77.6	22.1	0.3

^a^ Mattiske and Havel (1998).

^b^ Schoknecht, Tille, and Purdie (2004).

^c^ Terratree Pty Ltd. (2020).

### 2.2 Study Species and Initial Seed Viability and Germination Quantification

Six species native to Western Australia were selected for the study (Table 2) as they could replace some of the ecosystem functions and services lost due to the pathogen's impact on mid‐ and understorey species (Harshani, Tsakalos, et al. 2023). Four species *Acacia acuminata* , *Calothamnus sanguineus* , *Hakea laurina* and *Melaleuca seriata* are resistant to the pathogen (Harshani, McComb, et al. 2023). *Acacia saligna* is categorised as a *P. cinnamomi* tolerant host and *Banksia sessilis* as susceptible (Harshani, McComb, et al. 2023). The latter two species were included in the experiment to observe their field performance compared to the resistant species. *Acacia acuminata* and *A. saligna* seeds were treated in hot water at ca. 95°C for 2 min to break physical seed dormancy (Fryer 2006). The other four species have non‐dormant seeds and were left untreated. The viability of the seed batch of each species was determined using X‐ray analysis (Faxitron MX‐20 X‐ray cabinet, Tucson, Arizona, USA; Table 2). Seed weight was determined by weighing five replicates of 50 seeds (Table 2). The germination percentage of seeds under three incubation temperature combinations (18°C/7°C, 23°C/13°C, 32°C/17°C; 12 h/12 h light/dark) was assessed for 10 weeks. The incubator conditions of 18°C/7°C most closely approximate the temperatures prevailing at the time of germination of seeds in the field (Merritt et al. 2007). For the germination testing, seeds were first sterilised in a 2% (w/v) calcium hypochlorite (Ca(OCl)_2_) and washed three times with sterilised water. Four replicates of 25 seeds were then plated in 90 mm Petri dishes on an agar medium (0.7% w/v) and placed at each incubation temperature for each species.

**TABLE 2:** Seed characteristics of the six species used in the rehabilitation experiment.

Species	Family	Collection Date	Location of seed collection	Seed length (mm)	Mean seed weight (mg) ± SE	Viability (%)	Mean germination (%) at 18°C/7°C ± SE^a^	Mean germination (%) at 23°C/13°C ± SE^a^	Mean germination (%) at 32°C/17°C ± SE^a^
*Acacia acuminata*	Fabaceae	2021	Morawa, WA	5	9.9 ± 0.23	84.8 ± 2.42	99 ± 1.0	98 ± 2.0	100 ± 0
*Acacia saligna*	Fabaceae	2021	Woogenellup, WA	7	19.7 ± 0.20	92.4 ± 0.75	100 ± 0	100 ± 0	100 ± 0
*Calothamnus sanguineus*	Myrtaceae	2020	Yarrabee, WA	1	0.4 ± 0.00	89.7 ± 1.55	95 ± 1.9	77 ± 2.5	9 ± 3.4
*Melaleuca seriata*	Myrtaceae	2020	Wanneroo, WA	0.3	0.08 ± 0.00	91.1 ± 0.47	93 ± 1.0	91 ± 1.9	73 ± 4.4
*Banksia sessilis*	Proteaceae	2017	Wanneroo, WA	6	6.6 ± 0.19	91.6 ± 1.47	47 ± 5.9	43 ± 3.0	32 ± 2.8
*Hakea laurina*	Proteaceae	2021	WA	10	21.9 ± 0.28	92.4 ± 1.17	99 ± 1.0	92 ± 1.6	75 ± 9.0

Abbreviation: WA, Western Australia.

^a^ Mean final germination percentage observed over 10 weeks in incubators at three different temperatures (18°C/7°C, 23°C/13°C and 32°C/17°C; 12 h/12 h light/dark).

### 2.3 Seed Preparation for Extruded Pellets

Per species, the number of seeds used inside individual extruded pellets (hereafter referred to as ‘pellets’) varied based on the seed size (Table 3). All seeds used in the study except small seeds (i.e., *C. sanguineus* and *M. seriata* seeds) were x‐rayed, and only viable seeds that were uniform white grey in the x‐ray imagery were used. For small‐seeded species, it was impossible to x‐ray or efficiently count all the seeds needed for the total pellet requirements. Therefore, *C. sanguineus* and *M. seriata* seeds were weighed to give approximate samples of 23 and 22 seeds, which had 89% and 91% viability, respectively, to yield approximately 20 viable seeds per pellet. The use of different numbers of seeds according to seed size was based on the expectation that smaller seeds would show less vigour and survival than larger ones. As the objective was to establish at least one plant per pellet, monitoring focused on recording individual pellet performance (i.e., the number of pellets from which one or more seedlings emerged or survived, rather than the number of seedlings emerged from individual pellets).

**TABLE 3:** Pellet production method, number of seeds added and treatments applied for each species.

Species	Pellet production method	Number of viable seeds per pellet	Treatments		
			Non‐pelleted	Pelleted	
				Without additive	With additive
*Acacia acuminate*	Field‐deployed	5	+	+	+
*Acacia saligna*	Field‐deployed	5	+	+	+
*Calothamnus sanguineus*	Laboratory‐produced	20	+	+	+
*Melaleuca seriata*	Laboratory‐produced	20	+	+	+
*Banksia sessilis*	Laboratory‐produced	2	+	+	−
*Hakea laurina*	Laboratory‐produced	2	+	+	−

*Note:* For the *Acacia* species, the additive was a *Bradyrhizobium* sp., and for *C. sanguineus* and *M. seriata*, it was spores of *Pisolithus albus*, an ectomycorrhizal fungus.

### 2.4 Pellet Production Method

The pellet production method most suited to each species (Table 3) was determined from our previous work that compared seedling emergence from pellets produced in the laboratory (laboratory‐produced) or in the field (field‐deployed) (Harshani et al. 2024).

A slurry was prepared by mixing 750 g of bentonite (Bentonite Milled E; Bentonite Products WA Pty Ltd), 450 g of diatomaceous earth (Diatomite Fines < 0.6 mm; Mount Sylvia Diatomite Pty Ltd), 1800 g of sand (Silica, Hanson Australia Pty Ltd) with approximately 1500 g of water. Laboratory‐produced pellets were made using a rubber mould (56.5 cm × 36.3 cm, Matpro, Emu Plains, New South Wales) containing 200 hexagonal cavities. About 5 mL of the slurry was placed into each hexagonal cavity (each side 1.8 cm, 1 cm depth). Then, seeds were added to each cavity and mixed with the slurry using a steel spatula (150 mm long, 6 mm wide) to randomly place them within the pellet. The pellets were dried under fan‐forced air for 3 days before use in the field. Field‐deployed pellets were prepared in the field when setting up the experiment using a syringe with its tip removed to create an orifice of 3 cm diameter to deliver 5 mL of slurry (following methods like Stock et al. (2020)). The exact number of seeds was added to the slurry within the syringe and mixed using the steel spatula to ensure the seeds were randomly positioned. The slurry was extruded onto the soil surface by pushing the syringe plunger.

For pellet treatments containing a microbial additive, a preliminary glasshouse experiment was conducted using pellets with and without additives. These were the rhizobium bacterium (*Bradyrhizobium* spp.—WSM1741) for *A. acuminata* and *A. saligna* and spores of *Pisolithus albus*, an ectomycorrhizal fungus for *C. sanguineus* and *M. seriata* (Appendix S2). This preliminary assessment showed that seedlings emerging from the pellets formed rhizobium bacteria nodules and mycorrhizal fungi associations, respectively (Figure S2A,B). The presence of rhizobium nodules in *Acacia* spp. and mycorrhizal associations in *C. sanguineus* and *M. seriata* was recorded by observing the roots under the microscope. Additionally, the presence of *P. albus* was confirmed using DNA analysis. Based on this finding, pellets with and without additives were used for the field study for each of the four above species. For *A. acuminata* and *A. saligna* , about 0.3 and 3 g of *Bradyrhizobium* sp. inoculum grown in peat were added to the slurry when preparing the pellets. For *C. sanguineus* and *M. seriata* , 1.5 g of *P. albus* spore powder was added to the slurry. See Appendix S3 for detailed inoculum and spore preparation methods.

### 2.5 Experimental Design

**FIGURE 1:**
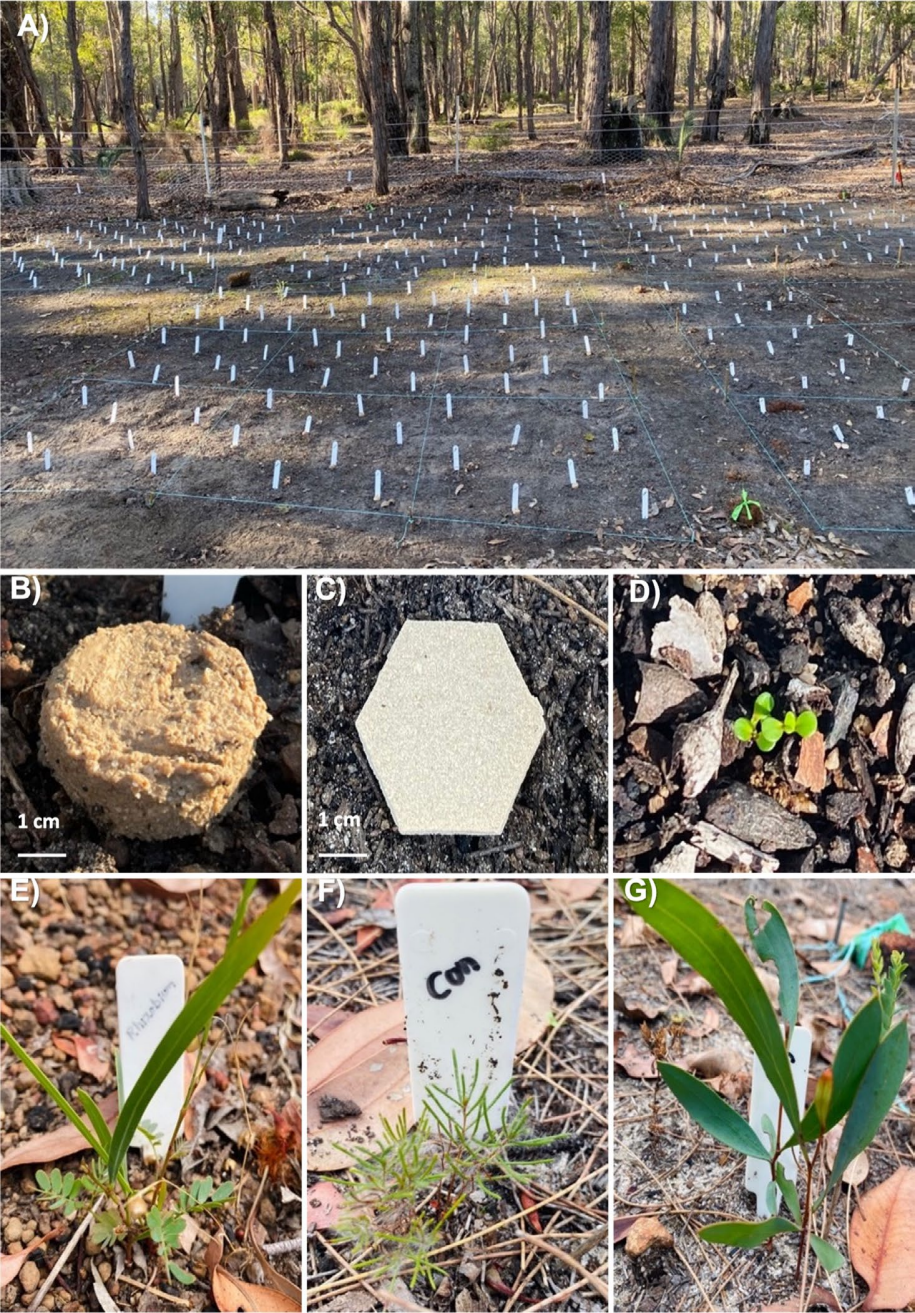
(A) A rehabilitation plot in Quail Street reserve after sowing pellets and non‐pelleted control seeds for six species. Different pellets used in the study: (B) field‐deployed pellets, (C) laboratory‐produced pellets, (D) *Melaleuca seriata* seedlings (6–8 weeks old after emergence). Surviving seedlings at 9 months after seeding, (E) *Acacia saligna* , (F) *Calothamnus sanguineus* and (G) *Hakea laurina*.

**FIGURE 2:**
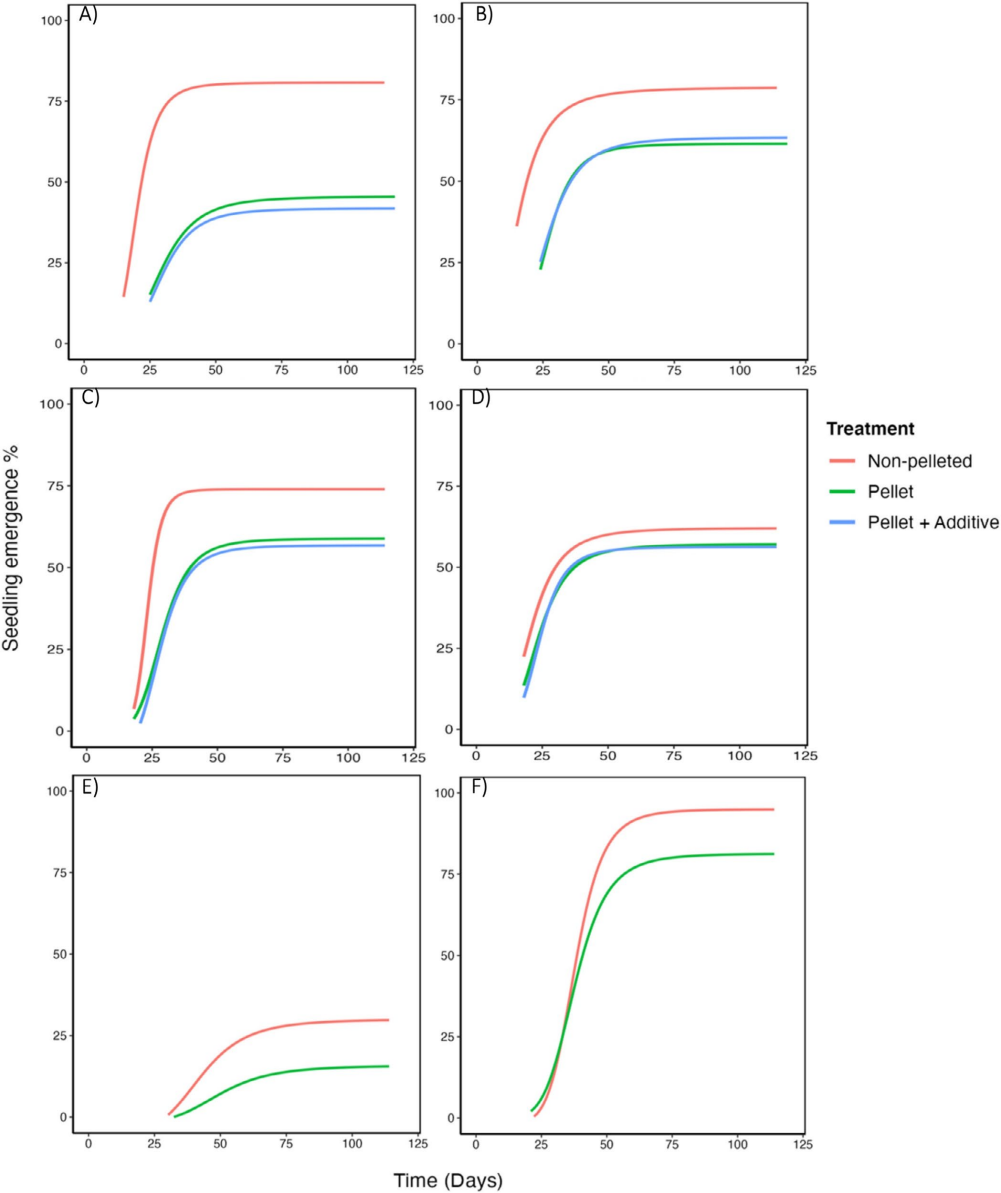
Cumulative seedling emergence of (A) *Acacia acuminata* , (B) *Acacia saligna* , (C) *Calothamnus sanguineus* , (D) *Melaleuca seriata* , (E) *Banksia sessilis* and (F) *Hakea laurina* from non‐pelleted seeds, pellets and pellets with inoculum of beneficial organisms over 120 days in the field.

A total of 18 rehabilitation plots (approximately 12 m × 8 m) were set up. There were six plots in *Phytophthora*‐infested areas in each of three reserves: Falls Road, Black Cockatoo and Quail Street (Figure 1A). A four‐strand wire fence with 90 cm of chicken netting (5 cm holes) at the base was erected around each plot to prevent seedling predation or damage by mammals. Within each plot, each species was sown in a separate subplot. Each rehabilitation plot had four 3 m × 2 m subplots for species with three treatments and two 2 m × 2 m subplots for species with two treatments (Table 3). A 0.5 m distance between subplots was maintained where possible with existing vegetation. Leaf litter in each subplot was raked off, and any existing plants were trimmed, leaving the roots. There were 24 replicates for each treatment giving 72 planting sites for species with three treatments (i.e., non‐pelleted precision buried seeds, pellets and pellet + additive) and 48 planting sites for species with two treatments (i.e., non‐pelleted precision buried seeds and pellets) in each subplot (Table 3). The treatments were randomised within each subplot. The pellets were placed on the soil surface (Figure 2B,C), and non‐pelleted seeds were buried 5 mm (half the thickness of the pellet) deep and covered with soil to stimulate precision planting.

No supplementary water was provided, and seedling emergence and survival depended on winter rain. Seedling emergence data were collected once a week for 2 months and then once every 2 weeks for an additional 2 months. The emergence of one or more seedlings from a pellet was recorded as ‘one’ for emergence. Seedling survival data were collected monthly for 5 months and then at 9 months after setting up the experiment. At the end of 9 months, the shoot height of each surviving seedling was recorded. When more than one seedling survived at a planting site, the mean height was calculated.

### 2.6 *Phytophthora cinnamomi* Isolation From Roots of Dying Seedling

Dying seedlings were collected at each observation time. Roots were washed with water to remove soil, then cut into pieces ~1–2 cm long and plated onto NARH medium (Simamora et al. 2018) to determine whether *P. cinnamomi* was present. At least five dying seedlings of each species in each reserve were plated.

### 2.7 Statistical Analysis

Statistical analysis was performed in R version 4.3.0 (R Core Team 2023). The means and standard errors for overall seedling emergence and survival percentages of each species in *Phytophthora*‐infested sites were calculated by combining all treatments using the *describeBy* function in the *psych* package (Revelle 2022). Seedling survival for each species was calculated as a percentage of emerged seedlings. A generalised linear mixed model (glm) for the presence–absence seedling emergence data and two linear mixed models (lme) for seedling survival and shoot growth data were performed for each species using *glmer* and *lmer* functions from the *lme4* package (Bates et al. 2015). In each glm and lme model, treatment and reserve were used as fixed effects and the plot was used as the random effect. To determine the best‐fitting model for each response variable Akaike's information criterion (AIC) values were used (Burnham and Anderson 2004). The assumptions of normality and homoscedasticity for each model were visually assessed. Analysis of variance (ANOVA) of these models was performed to determine whether there was a significant difference between treatments and reserves. When there was a significant difference, post hoc Tukey analyses were performed using the *emmeans* function from *emmeans* package (Lenth 2023). Results from the models were visually presented using the dose–response curve (*drc*) package and *ggplot2* package (Ritz et al. 2015; Wickham 2016).

## 3 Results

### 3.1 Seedling Emergence

When data from all the treatments were combined, the highest seedling emergence after 4 months was observed in *H. laurina*, which was 88.4 ± 3.43% (Table 4). The remaining resistant or tolerant species also resulted in more than 50% seedling emergence (Table 4). However, *B. sessilis* , the only susceptible species used in the study had only 22.8 ± 1.57% seedling emergence (Table 4).

**TABLE 4:** Mean and standard error of percentage seedling emergence (after 4 months: end of spring) and percentage of emerged seedlings that survived (after 9 months: end of summer drought period), for each species with all treatments combined.

Species	Seedling emergence % (mean ± SE)	Seedling survival % (mean ± SE)
*Acacia acuminata*	55.9 ± 3.68	25.5 ± 3.12
*Acacia saligna*	68.4 ± 5.21	16.3 ± 2.58
*Banksia sessilis*	22.8 ± 1.57	36.9 ± 6.02
*Calothamnus sanguineus*	63.2 ± 5.73	53.0 ± 2.28
*Hakea laurina*	88.4 ± 3.43	83.6 ± 2.82
*Melaleuca seriata*	58.6 ± 3.91	0

When different treatments were compared, the seedling emergence from *M. seriata* pellets (with or without an additive) was similar (*p* ≥ 0.05; Table S4; Figure 2) to that of non‐pelleted buried seeds. For the remaining species, the seedling emergence significantly (*p* ≤ 0.001) varied between treatments (Table S5) and non‐pelleted seeds had the highest emergence compared to pellets (with or without an additive; Table S4; Figure 2). Although seedling emergence from pellets was generally lower than from non‐pelleted buried seeds, the reduction was less than 18% for all the species, except for *A. acuminata* , which it was 36% lower (Table S6). The presence of either rhizobium bacteria or ectomycorrhizal fungus as an additive in the pellet did not significantly (*p* ≥ 0.05) affect seedling emergence (Table S4).

The lme analysis also showed that the seedling emergence of most of the species varied between reserves (*p* ≤ 0.001; Table S7). Among the three reserves, seedling emergence of all the treatments for *A. acuminata* and *A. saligna* was significantly (*p* ≤ 0.001) lower in Black Cockatoo reserve and for *C. sanguineus* , *M. seriata* and *H. laurina*, it was significantly (*p* ≤ 0.05) higher in Falls Road reserve (Table S8). No significant (*p* ≥ 0.05) difference in seedling emergence between reserves was observed in *B. sessilis* (Table S7). The complete model outputs are given in Table S9.

### 3.2 Seedling Survival After 9 Months

Among the resistant species, *H. laurina* showed the highest seedling survival (83.6 ± 2.82%) after 9 months when data from all the treatments were combined (Table 4). Other resistant species, *A. acuminata* (25.5 ± 3.12%) and *C. sanguineus* (53.0 ± 2.28%), also exhibited substantial seedling survival except for *M. seriata* where no seedlings survived after 9 months (Table 4). *Acacia saligna* had a low seedling survival of 16.3 ± 2.58% and the only susceptible species, *B. sessilis* , showed 36.9 ± 6.02% seedling survival (Table 4).

**FIGURE 3:**
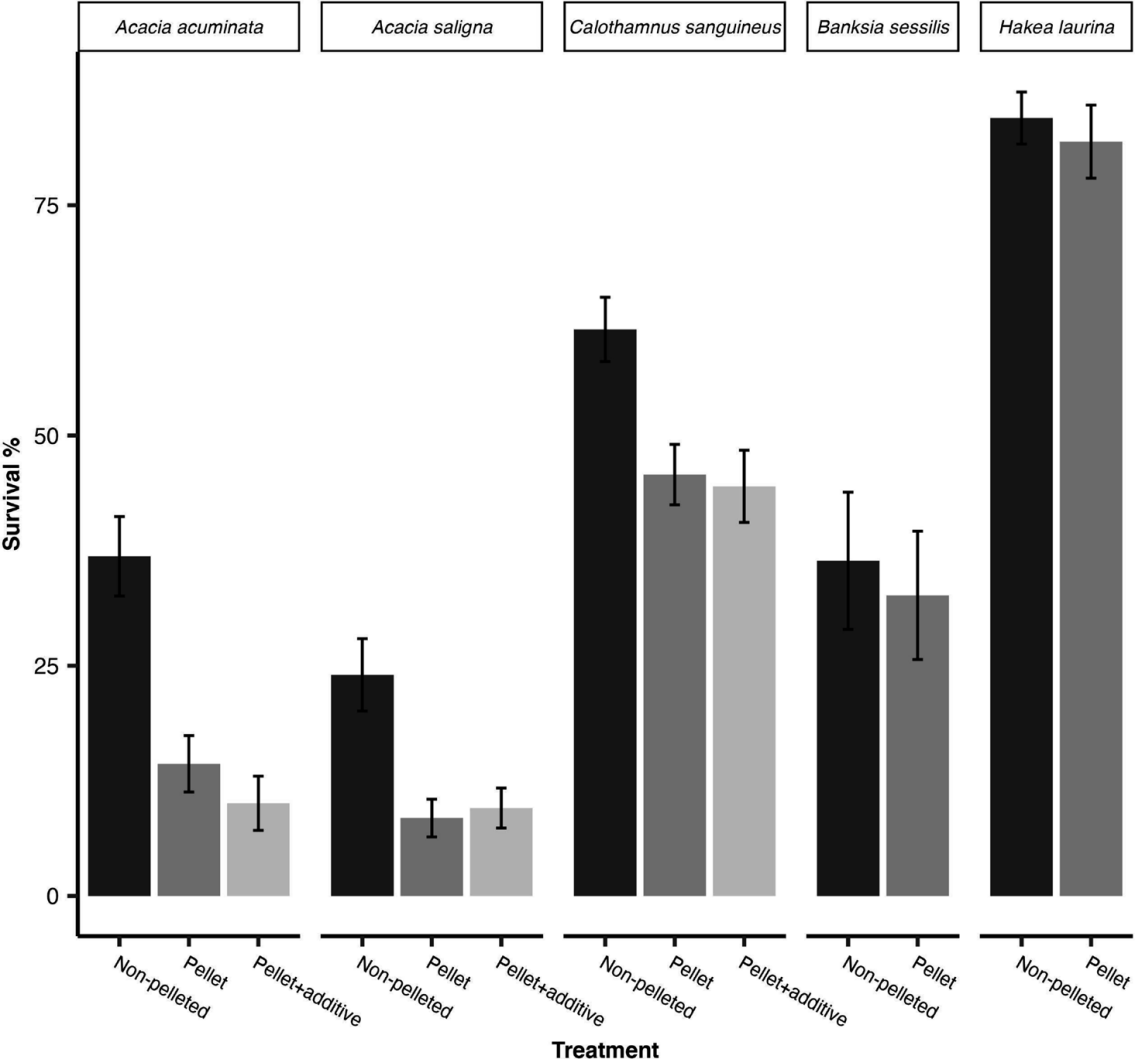
Seedling survival percentages of *Acacia acuminata* , *Acacia saligna* , *Calothamnus sanguineus* , *Melaleuca seriata, Banksia sessilis* and *Hakea laurina*. The latter two species had only two treatments (i.e., non‐pelleted seeds and pellets).

After 9 months, when comparing different treatments, the seedling survival of *B. sessilis* and *H. laurina* pellets was similar to that of non‐pelleted buried seeds (*p* ≥ 0.05; Table S4; Figure 3). For the remaining species, seedling survival varied significantly (*p* ≤ 0.01) between treatments (Table S5). Non‐pelleted buried seeds of *A. acuminata* (36.9 ± 4.32%), *A. saligna* (24.0 ± 3.92%) and *C. sanguineus* (61.5 ± 3.49%) showed significantly (*p* ≤ 0.01) higher seedling survival compared to both pellets and pellets with an additive (Figure 3, Table S4). The presence of an additive in the pellet, rhizobium bacteria or ectomycorrhizal fungus did not significantly (*p* ≥ 0.05) affect the seedling survival of any of the species (Table S4).

Seedling survival of all species did not vary significantly (*p* ≥ 0.05) between reserves (Table S7) except for *B. sessilis* . The survival of *B. sessilis* seedlings from all the treatments was significantly (*p* ≤ 0.05) lower in the Quail Street reserve compared to the other two reserves (Table S8). The complete model outputs are given in Table S9.

### 3.3 Seedling Shoot Growth

**FIGURE 4:**
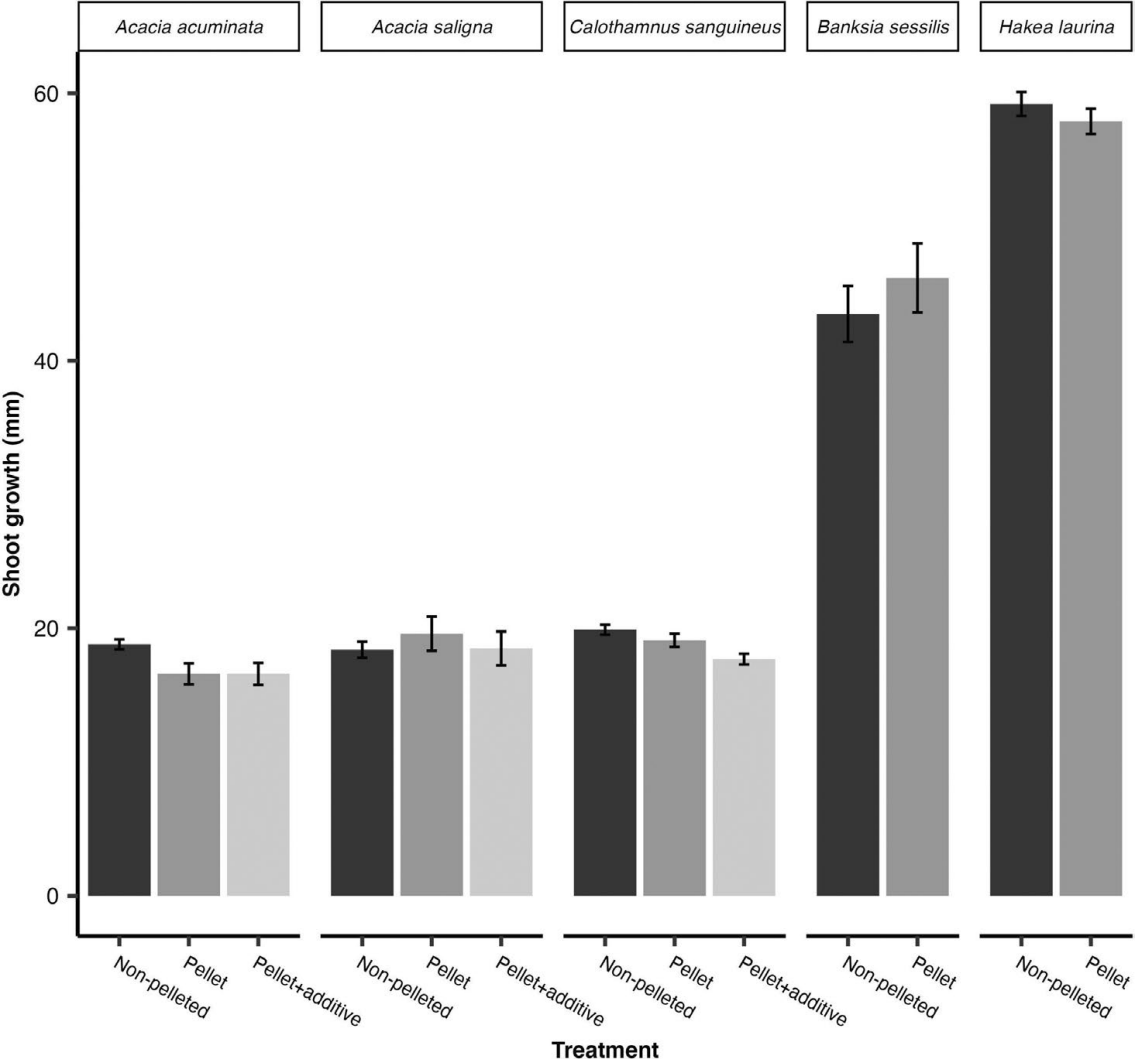
Seedling shoot heights of *Acacia acuminata* , *Acacia saligna* , *Calothamnus sanguineus* , *Melaleuca seriata* , *Banksia sessilis* and *Hakea laurina*.

At 9 months, *A. saligna, B. sessilis* and *H. laurina* seedlings of all treatments had similar (*p* ≥ 0.05) shoot growth (Figure 4, Table S5). A significantly (*p* ≤ 0.01) higher shoot growth was observed in non‐pelleted seedlings of *A. acuminata* (18.8 ± 0.37 mm) compared to seedlings from pellets (16.6 ± 0.78 mm) and pellets with an additive (16.6 ± 0.82 mm; Table S4). In *C. sanguineus* , shoot growth of non‐pelleted and pelleted seedlings was the same, but both were significantly (*p* ≤ 0.01) higher than seedlings from pellets with an additive (Table S4).

Shoot growth of all species did not significantly (*p* ≥ 0.05) vary between reserves except for *A. acuminata* and *H. laurina* (Table S7) where shoot growth was significantly (*p* ≤ 0.001) lower in the Black Cockatoo reserve and Quail Street reserve, respectively (Table S8). The complete model outputs are given in Table S9. Overall, seedling emergence, survival and shoot growth were lower on the Black Cockatoo and Quail Street reserves.

### 3.4 Causes of Seedling Deaths

No *P. cinnamomi* was isolated from the roots of dying seedlings of any species. Most dying seedlings showed drought symptoms. Severe insect damage was observed on *Acacia* seedlings, with all leaves frequently eaten. However, in some cases, the cause of death could not be confirmed as the seedlings ‘disappeared’ between assessments.

## 4 Discussion

This study indicated the feasibility of rehabilitating *P. cinnamomi*‐infested sites with native resistant species, a topic that has been poorly investigated to date. It also demonstrated that pellets can effectively facilitate the distribution of seeds of various sizes and shapes. All the species emerged in varying percentages in *Phytophthora*‐infested sites, regardless of each species' susceptibility or resistance to the pathogen. However, seedling emergence of *B. sessilis* , a species susceptible to *P. cinnamomi*, was the lowest and only half that observed under laboratory conditions (Tables 2 and 4). This could be due to the impact of *P. cinnamomi* on seed germination and seedling emergence of this species.

For all species except *M. seriata* , seedling emergence from pelleted seeds was slightly lower than precision buried seeds. Despite this reduction, seedling emergence from the pellets remained within an acceptable range for establishing these species in *Phytophthora*‐infested sites. This suggests that using pellets are a viable alternative to precision seed placement via direct seeding machinery for *Phytophthora*‐infested rehabilitation projects.

The lower seedling emergence obtained from the pellets in some species may have been due to the early disintegration of the pellets. Generally, pellets should retain their structure to provide a microsite favourable for seed germination and seedling emergence (Erickson et al. 2021). However, in the present study, most pellets disintegrated early due to heavy winter rainfall, leaving ungerminated seeds exposed on the soil surface. These seeds were then subjected to desiccation and predation by birds and insects, resulting in low seedling emergence. Conversely, non‐pelleted seeds that were manually buried, similar to what would be expected from precision seeding machines, showed higher seedling emergence than pellets. These findings contrast with other studies where pellets resulted in higher seedling emergence in glasshouse (Madsen et al. 2016) and field studies (Ritchie, Stevens, and Erickson 2020) compared to buried control seeds. The species that showed no significant difference in seedling emergence between non‐pelleted seeds and pelleted seeds was *M. seriata* , which had the smallest seeds. After the disintegration of the pellets, these small seeds may be unattractive to predators or washed into soil cavities.

After 9 months, a substantial percentage of seedlings survived from two *P. cinnamomi*‐resistant species, *C. sanguineus* and *H. laurina*. In ecosystems with a Mediterranean climate, the survival of most emerging seedlings is restricted by the prolonged summer drought (Padilla and Pugnaire 2007). In the present study, most seedling deaths in all the species appeared to be due to a lack of rainfall between December 2022 and February 2023 (Australian Bureau of Meteorology 2023). In the future, selecting species resistant to *P. cinnamomi* and tolerant to drought may result in better seedling establishment. Although *A. acuminata* is resistant to the pathogen and *A. saligna* is a tolerant host (Harshani, McComb, et al. 2023), the seedling survival of both species was low. This is probably due to the severe insect damage observed in *Acacia* seedlings, although fencing protected them from mammalian predators. Previous reports also suggest that *Acacia* species are prone to foliage damage by insects (Searle 1997; Wingfield, Roux, and Wingfield 2011). Seed pellets prepared (or seedlings sprayed) with insecticides could be tested in future studies to reduce insect damage and increase seedling survival. None of the *M. seriata* seedlings survived after 9 months, even though the species is resistant to the pathogen (Harshani, McComb, et al. 2023). Possibly this small‐seeded species did not develop a root system sufficient to cope with harsh summer drought compared to large‐seeded species (Macera, Pereira, and Souza 2017). Despite being susceptible to the pathogen, when all treatments were combined, around 30% of *B. sessilis* seedlings survived, likely due to disease escape. A possible explanation for lower shoot growth observed in seedlings of *A. acuminata* and *C. sanguineus* from pelleted seeds, compared with non‐pelleted ones, could be due to the early disintegration of the pellets, leaving the seeds exposed on the soil surface and possibly slower to develop a deep root system compared to the precision buried non‐pelleted seeds. Incorporating either rhizobium bacteria or ectomycorrhizal fungal spores into the pellet did not improve seedling survival or shoot growth. This may be because the soil on the sites had adequate natural inoculum of these microbes for the control plants despite the presence of *P. cinnamomi*. There appear to be a few other studies of the effect of symbiotic organisms added to pellets of native species. Dadzie et al. (2022) included cyanobacteria in extruded pellets of *Acacia inaequilatera* and *Triodia epactia* and obtained higher seedling emergence in a study on an iron ore mine site in the Pilbara, Western Australia, with the provision of irrigation to stimulate typical arid rainfall events. Seedling survival in the jarrah forest was best where there was more vegetation, as observed at Falls Road reserve. This correlates with other studies in which seedling establishment was higher in areas with some protective vegetation than in open areas (Becerra, Aqueveque, and Velasco 2022).

There are a few limitations associated with the present study. Comparable plots of pelleted and non‐pelleted seeds in areas where the pathogen is absent could not be included due to limited project resources. Additionally, the current trial was established in mid‐winter (i.e., July). Sowing in late autumn or early winter might have allowed the development of a more extensive root system before the onset of the summer, potentially improving survival.

Further research is needed to improve the pellet formulation to achieve slow disintegration of the pellets until seeds germinate and emerge. The best pellet composition may be different for large and small seeds. Pellet formulations with insecticides should also be tested to reduce insect predation. Although a glasshouse study showed that *Acacia* seeds germinated more successfully from field‐deployed pellets than from those prepared in the laboratory, it was laborious and time‐consuming to produce the pellets in the field and this method is not applicable to large‐scale applications. Modification of the pellet composition to be more suitable for *Acacia* is required. Further investigation is also required on site preparation. The cost benefit of fencing the sites before seeding needs to be assessed as pelleting is expected to reduce seed predation, and the amount of damage from native mammals has not been quantified. There could be considerable benefit of rehabilitating after a control burn as this could reduce insect predation (Whelan and Main 1979), and possibly mammalian herbivore predation. The possibility of whether pelleted seed may also be effective for revegetation of black gravel sites with very little remaining vegetation due to the severe *P. cinnamomi* impact should be investigated compared to the sites in the forest investigated here.

This study demonstrated that *Phytophthora*‐resistant native species can be successfully established in *Phytophthora*‐infested areas. Pelleting facilitated the distribution of seeds of resistant species with different seed shapes and sizes and resulted in seedling emergence and survival proportions only slightly slower than that of precision buried (non‐pelleted) seeds, despite the early disintegration. Including microbes as an additive did not enhance seedling emergence, survival or growth in the present study. However, modifying the pellet composition to delay disintegration in the field could increase seedling emergence and survival to equal that of precision buried seeds. These findings suggest that, for rehabilitation projects on *Phytophthora*‐infested sites, using pellets is a viable alternative to precision seed placement using direct seeding machinery.

## Author Contributions

**Himbutugoda S. Harshani:** conceptualization (supporting), data curation (lead), formal analysis (lead), investigation (lead), methodology (lead), writing – original draft (lead), writing – review and editing (lead). **Todd E. Erickson:** conceptualization (supporting), investigation (supporting), methodology (supporting), supervision (lead), writing – review and editing (supporting). **Jen McComb:** conceptualization (supporting), writing – original draft (supporting), writing – review and editing (supporting). **Treena Burgess:** conceptualization (supporting), funding acquisition (supporting), methodology (supporting), supervision (supporting), writing – review and editing (supporting). **Giles Hardy:** conceptualization (lead), funding acquisition (lead), methodology (supporting), supervision (lead), writing – review and editing (supporting).

## Conflicts of Interest

The authors declare no conflicts of interest.

## Supporting information


Appendix S1.

## Data Availability

The data that support the findings of this study are openly available in Zenodo public data repository at https://doi.org/10.5281/zenodo.14538632.
