# Supplementary material for: Seed‐Based Rehabilitation of Phytophthora cinnamomi‐Infested Forest Sites
Source: Ecol Evol. 2025 Feb 24;15(2):e70900. doi: 10.1002/ece3.70900 (PMC11850445; doi:10.1002/ece3.70900)
Supplement: Supplementary file 1 — Appendix S1. [file ECE3-15-e70900-s001.docx]

**Supplementary Materials**

**S1 – Environmental conditions (rainfall, temperature) during the trial period**

**Figure S1** Rainfall, mean minimum and maximum temperatures in Mundaring, Western Australia, from July 2022 to April 2023 (Australian Bureau of Meteorology 2023)

**S2 - Preliminary glasshouse experiment**

**Aim**

To determine whether *Acacia acuminata* and *A. saligna*, form nodules of rhizobium when bacteria inoculum (*Bradyrhizobium* spp.) is provided in a pellet, and if *Calothamnus sanguineus* and *Melaleuca seriata* form mycorrhizal associations when provided with spores of *Pisolithus albus*, an ectomycorrhizal fungus, in a pellet.

**Experimental design**

A slurry was prepared by mixing bentonite, diatomaceous earth, sand in a ratio of 25:15:60 and water as required. Two types of pellets: pellets with and without an additive, were then prepared using a rubber mould (56.5 cm x 36.3 cm, Matpro, Emu Plains, New South Wales). The number of seeds added to the pellets varied based on the species: 5 seeds were added for *Acacia acuminata* *A. saliga* while 20 seeds were added for *Calothamnus sanguineus* and *Melaleuca seriata*. For pellets with additive, about 0.3g of *Bradyrhizobium* sp. inoculum grown in peat was mixed into the slurry for *A. acuminata* and 3g for *A. saligna.* Additionally, 1.5g of *Pisolithus albus* spore powder was added to the slurry when preparing pellets for *Calothamnus sanguineus* and *Melaleuca seriata*. Control pellets were prepared without any added beneficial organisms.

Plastic pots (69 mm, 0.3 L, Garden City Plastics, Forrestfield, Western Australia) were filled with sand (Silica, Hanson Australia Pty Ltd). One pellet of each type was placed on the sand surface of each pot. There were 20 replicate pots for each species, 10 for control pellets and 10 for pellets with an additive. Pots were watered using sprinklers after sowing the pellets, and then twice a day for 5 mins. After 18 weeks seedlings examined for nodule formation and mycorrhizal fungi associations.

**Confirmation of the presence of *P. albus* in mycorrhizal associations**

Roots with mycorrhizal associations were collected under the microscope for both species. There were three root samples for each species, two from pellets inoculated with *P. albus* and one from non-inoculated pellets. DNA was extracted from the roots using the Qiagen DNeasy Plant Pro Kit as per manufacture’s protocol. The ITS2 region was then amplified using the fungal specific primer fITS7 (Integrated DNA Technologies, Baulkham Hills, New South Wales, 2153), and the eukaryotic primer ITS4 (Integrated DNA Technologies, Baulkham Hills, New South Wales, 2153) (Ihrmark et al., 2012). PCR products were sent to Australian Genome Research Facility (AGRF).

Sequence deconvolution (of forward reads only) such as quality control and clustering was carried out using VSEARCH ([https://github.com/](https://aus01.safelinks.protection.outlook.com/?url=https%3A%2F%2Fgithub.com%2F&data=05%7C01%7CShanika.Harshani%40murdoch.edu.au%7C33bc1609da0f440b87ca08db8755fe44%7Cc00d4c1bcf7b4e93b7c710113a9bc230%7C1%7C0%7C638252576793122878%7CUnknown%7CTWFpbGZsb3d8eyJWIjoiMC4wLjAwMDAiLCJQIjoiV2luMzIiLCJBTiI6Ik1haWwiLCJXVCI6Mn0%3D%7C3000%7C%7C%7C&sdata=5tMo0BSK5p1juCrnYmuZ7Rwo7ur5LCz0mmw3rLN8Wt8%3D&reserved=0)torognes/vsearch.wiki.git). Specifically, sequences less than 150 bp and low mean quality (<20) were removed. Sequences that passed quality control were clustered into operational taxonomic units (OTUs). OTUs were identified by searching against the public database provided by UNITE (Abarenkov et al., 2022). Identities were further checked by blasting against the NCBI nucleotide collection (nr/nt) database to identify closely related taxa at the lowest taxonomic resolution possible.

**Results and discussion**

Rhizobium bacteria nodules were observed in 70% and 80% of seedlings from pellets with bacteria inoculum in both *A. acuminata* and *A. saligna* and no nodules were observed in seedlings from control pellets showing the necessity of inoculation for nodule formation. In *C. sanguineus* and *M. seriata*, 100% and 80% of seedlings, respectively, from pellets with fungal spores developed mycorrhizal associations. However, about 40% of *M. seriata* and 70% of *C. sanguineus* control pellet seedlings also developed mycorrhizal associations. This suggests that the sand used in the experiment may have included some inoculum, or the fungi were present as glasshouse contaminants.

DNA analysis revealed that *P. albus* was present in both root samples of plants from inoculated pellets in *C. sanguineus* and *M. seriata*. However, in the *C. sanguineus* control*,* *P. albus* and *Setophoma terrestris* were recorded whereas the *M. seriata* control did not record any fungi species (Table S2).

i)


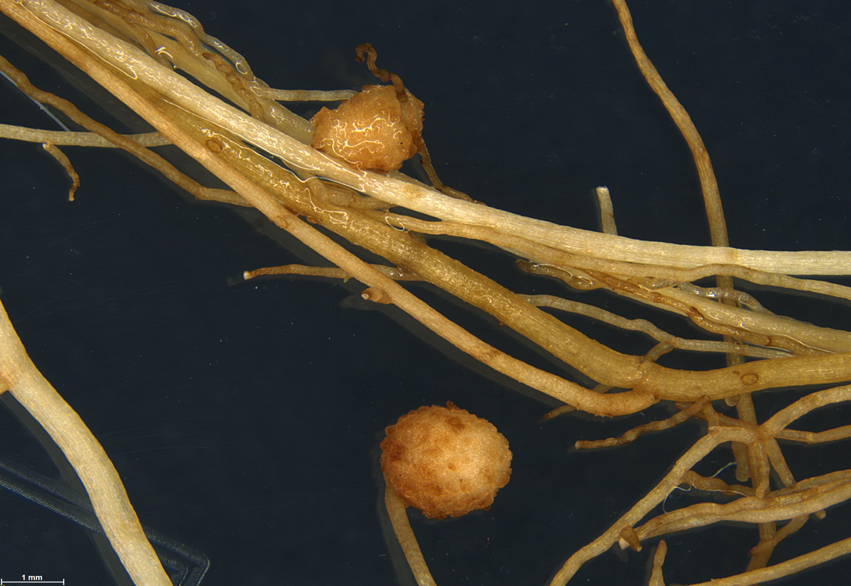

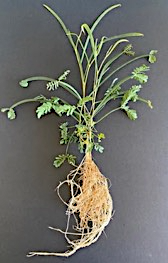


ii))


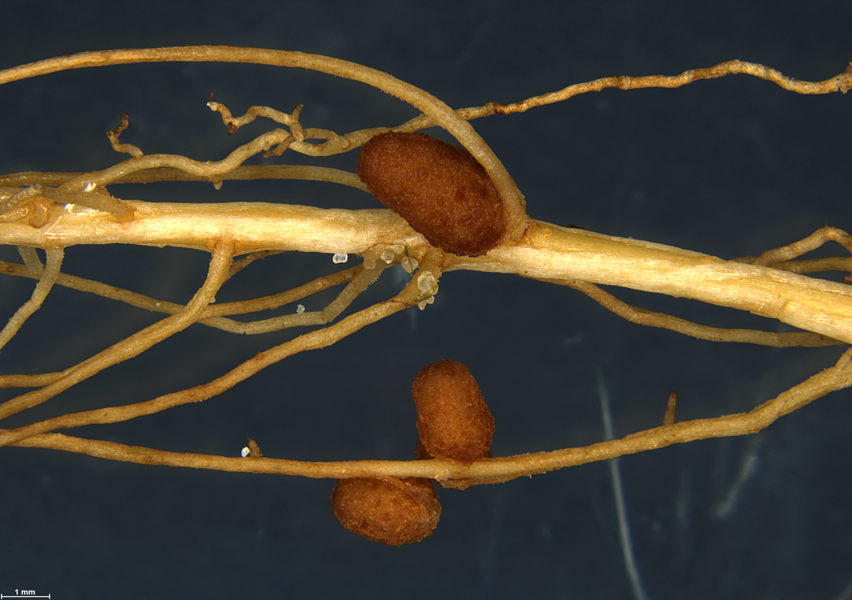

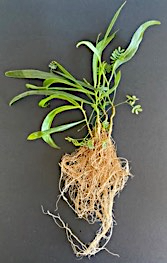


**Figure S2 A)** Bacteria nodules in i) *Acacia acuminata* ii) *Acacia saligna* roots on seedlings from pellets that included rhizobium in the glasshouse preliminary experiment.


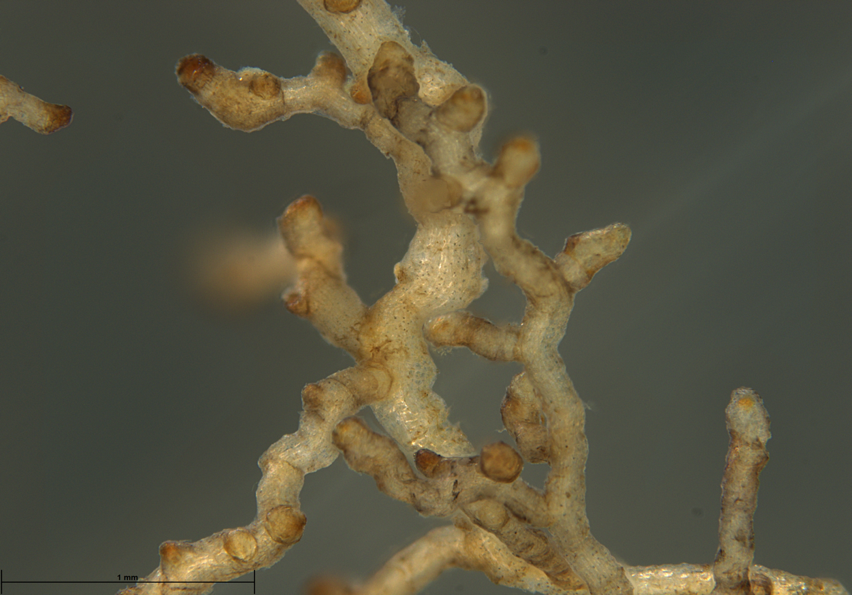


i)


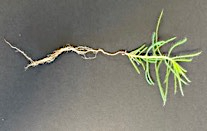

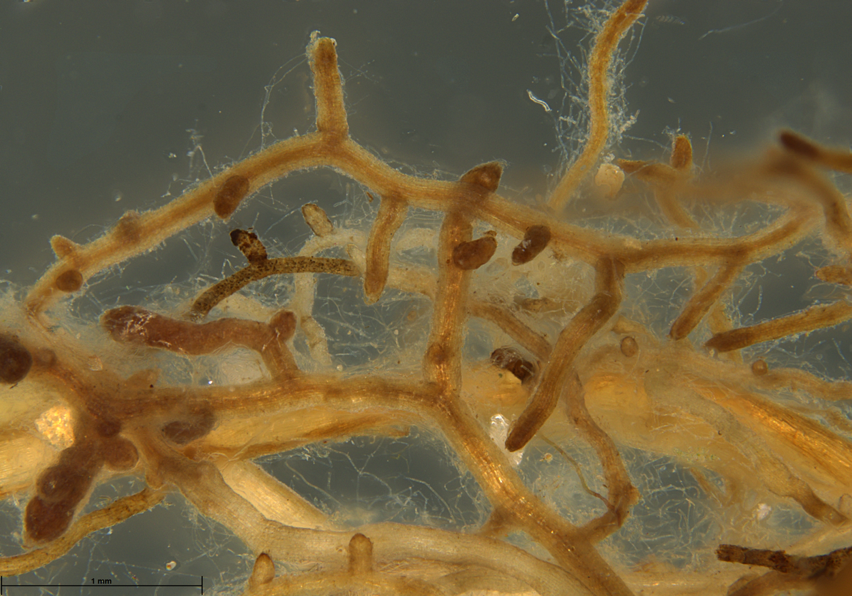


ii)


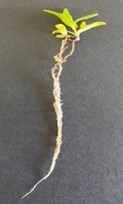


**Figure S2 B)** Mycorrhizal fungi observed in i) *Calothamnus ssanguineus* ii) *Melaleuca seriata* roots on seedlings from pellets that included mycorrhizal fungal spores in the glasshouse preliminary experiment.

**Table S2** Fungal species recoded in the root samples of *Melaleuca seriata* and *Calothamnus sanguineus*. Presence and absence of recorded species are shown.

| **Organism** | ***Melaleuca seriata*** | | ***Calothamnus sanguineus*** | |
| --- | --- | --- | --- | --- |
|  | Control | Inoculated | Control | Inoculated |
| *Pisolithus albus* | − | + | + | + |
| *Scleroderma* sp. | − | − | − | + |
| *Setophoma terrestris* | − | − | + | − |

**S3 – Inoculum and spore preparation methods**

**Rhizobium inoculum preparation**

Cultures of *Bradyrhizobium* spp. WSM1741 (Yates et al., 2004) were obtained from suspensions of ampouled stock plated onto ½ LA media (Hungria et al., 2016) and incubated at 28°C. Single colonies were subbed into 5 ml of ½ LA broth, shaken at 250 rpm and again incubated at 28°C. A 5 mL aliquot of culture was subbed into 80 ml of ½ LA broth in a 250 ml conical flask, shaken at 200 rpm at 28°C until it reached a concentration of 10^7^ cells ml^-1^. The number of viable cells in broth culture was counted using the Miles and Misra drop plate counting method (Miles et al., 1938).

A packet (approx. 160g) containing pre-sterilised irradiated peat was wiped with 70% ethanol. Using a sterile syringe and a needle 60ml of broth culture from the conical flask was injected into the peat packet. The injection site was cleaned with 70% ethanol and covered with with clear acrylic packing tape. The peat packet was massaged carefully to evenly distribute the broth culture within the packet, and it was incubated at 28°C for 12 days. The number of viable cells in this peat inoculant was counted using Miles and Misra method and showed count of 10^8^ cells g^-1^ of peat.

Based on seed weight (*Acacia acuminata*, 9.9 ± 0.23mg; *A. saligna*, 19.7 ± 0.2mg), 10,000 and 100,000 cells were required per seed to ensure an adequate population for nodulation (Deaker et al., 2016). Around 0.1g and 1g of peat inoculum were required to inoculate 720 seeds of *A. acuminata* and *A. saligna*, respectively. As the inoculum was added to the slurry, to ensure that each seed received the recommended number of cells, three times the required amount of peat inoculant was used (i.e., 0.3g peat inoculant for *A. acuminata* and 3g for *A. saligna*) to prepare pellets.

**Mycorrhizal fungi spore preparation**

Fruit bodies of ectomycorrhizal fungi, *Pisolithus* spp. were collected from the Murdoch University grounds (Perth, Western Australia). The fruiting bodies were cleaned with a fine paintbrush to remove soil and dirt and then cut into halves and placed in paper bags. The fruiting bodies in paper bags were dried at 30°C for 48 h. Dried fruiting bodies were then crushed by hand, sieved (700μm) and stored at 25°C (Chen et al., 2006). Before pellet preparation, the number of viable spores in 0.1g spore powder was counted using a dilution series (from 10^-1^ to 10^-5^) and a haemocytometer. About 10^7^ spores were required per pellet. To ensure that each seed received enough spores, three times of the required amount of spore powder was used for each species (i.e., 1.5g of spore powder for both *C. sanguineus* and *M. seriata*).

**Table S4** P values for the pairwise comparisons of seedling emergence, survival, and shoot growth between non-pelleted and pellets or pellets with inoculum of beneficial organisms using estimated marginal means for generalised linear mixed model and linear mixed model in six species.

| **Response** | **Species** | **Comparison** | **Estimate** | **Standard Error** | **t / z ratio** | **p value** |
| --- | --- | --- | --- | --- | --- | --- |
| Emergence | *A. acuminata* | (Non-pelleted) – Pellet | 1.78 | 0.17 | 10.76 | **<0.001** |
|  |  | (Non-pelleted) – (Pellet + Additive) | 1.94 | 0.17 | 11.63 | **<0.001** |
|  |  | Pellet – (Pellet + Additive) | 0.16 | 0.15 | 1.08 | 0.53 |
|  | *A. saligna* | (Non-pelleted) – Pellet | 1.11 | 0.18 | 6.35 | **<0.001** |
|  |  | (Non-pelleted) – (Pellet + Additive) | 1.02 | 0.18 | 5.84 | **<0.001** |
|  |  | Pellet – (Pellet + Additive) | -0.09 | 0.16 | -0.55 | 0.85 |
|  | *C. sanguineus* | (Non-pelleted) – Pellet | 0.86 | 0.16 | 5.37 | **<0.001** |
|  |  | (Non-pelleted) – (Pellet + Additive) | 0.95 | 0.16 | 5.93 | **<0.001** |
|  |  | (Pellet + Additive) – Pellet | -0.09 | 0.15 | -0.59 | 0.82 |
|  | *M. seriata* | (Non-pelleted) – Pellet | 0.19 | 0.14 | 1.34 | 0.37 |
|  |  | (Non-pelleted) – (Pellet + Additive) | 0.23 | 0.14 | 1.62 | 0.24 |
|  |  | (Pellet + Additive) – Pellet | -0.04 | 0.14 | -0.28 | 0.96 |
|  | *B. sessilis* | (Non-pelleted) – Pellet | 0.81 | 0.17 | 4.79 | **<0.001** |
|  | *H. laurina* | (Non-pelleted) – Pellet | 1.72 | 0.27 | 6.31 | **<0.001** |
| Survival | *A. acuminata* | (Non-pelleted) – Pellet | 22.54 | 4.62 | 4.88 | **<0.001** |
|  |  | (Non-pelleted) – (Pellet + Additive) | 26.81 | 4.62 | 5.80 | **<0.001** |
|  |  | Pellet – (Pellet + Additive) | 4.27 | 4.62 | 0.93 | 0.63 |
|  | *A. saligna* | (Non-pelleted) – Pellet | 15.55 | 3.74 | 4.16 | **<0.001** |
|  |  | (Non-pelleted) – (Pellet + Additive) | 14.46 | 3.74 | 3.87 | **<0.01** |
|  |  | Pellet – (Pellet + Additive) | -1.09 | 3.74 | -0.29 | 0.95 |
|  | *C. sanguineus* | (Non-pelleted) – Pellet | 15.77 | 5.93 | 3.13 | **<0.01** |
|  |  | (Non-pelleted) – (Pellet + Additive) | 17.05 | 5.93 | 3.39 | **<0.01** |
|  |  | Pellet – (Pellet + Additive) | 1.27 | 5.93 | 0.25 | 0.96 |
|  | *B. sessilis* | (Non-pelleted) – Pellet | 3.76 | 8.72 | 0.43 | 0.66 |
|  | *H. laurina* | (Non-pelleted) – Pellet | 2.57 | 4.19 | 0.61 | 0.54 |

**Table S4** continued.

| **Response** | **Species** | **Comparison** | **Estimate** | **Standard Error** | **t / z ratio** | **p value** |
| --- | --- | --- | --- | --- | --- | --- |
| Shoot growth | *A. acuminata* | (Non-pelleted) – Pellet | 2.70 | 0.85 | 3.19 | **<0.01** |
|  |  | (Non-pelleted) – (Pellet + Additive) | 3.03 | 0.92 | 3.29 | **<0.01** |
|  |  | Pellet – (Pellet + Additive) | 0.33 | 1.10 | 0.95 | 0.95 |
|  | *A. saligna* | (Non-pelleted) – Pellet | -0.92 | 1.36 | -0.67 | 0.78 |
|  |  | (Non-pelleted) – (Pellet + Additive) | 0.37 | 1.34 | 0.28 | 0.96 |
|  |  | Pellet – (Pellet + Additive) | 1.29 | 1.61 | 0.79 | 0.70 |
|  | *C. sanguineus* | (Non-pelleted) – Pellet | 1.04 | 0.57 | 1.83 | 0.16 |
|  |  | (Non-pelleted) – (Pellet + Additive) | 2.17 | 0.57 | 3.81 | **<0.01** |
|  |  | Pellet – (Pellet + Additive) | -1.13 | 0.64 | -1.78 | 0.17 |
|  | *B. sessilis* | (Non-pelleted) – Pellet | -2.48 | 3.92 | -0.63 | 0.53 |
|  | *H. laurina* | (Non-pelleted) – Pellet | 1.6 | 1.25 | 1.28 | 0.20 |

**Table S5** Results from generalised linear mixed model and linear mixed model ANOVA analysis for the effect of three different treatments on seedling emergence, survival, and shoot growth of six species.

| **Response** | **Species** | **Df** | **Chi sq / F value** | **P value** |
| --- | --- | --- | --- | --- |
| Emergence | *A. acuminata* | 2 | 155.44 | **<0.001** |
|  | *A. saligna* | 2 | 46.96 | **<0.001** |
|  | *C. sanguineus* | 2 | 41.41 | **<0.001** |
|  | *M. seriata* | 2 | 2.97 | 0.22 |
|  | *B. sessilis* | 1 | 22.98 | **<0.001** |
|  | *H. laurina* | 1 | 39.79 | **<0.001** |
| Survival | *A. acuminata* | 2 | 19.44 | **<0.001** |
|  | *A. saligna* | 2 | 10.79 | **<0.001** |
|  | *C. sanguineus* | 2 | 7.12 | **<0.01** |
|  | *B. sessilis* | 1 | 0.19 | 0.66 |
|  | *H. laurina* | 1 | 0.38 | 0.54 |
| Shoot growth | *A. acuminata* | 2 | 8.98 | **<0.001** |
|  | *A. saligna* | 2 | 0.35 | 0.70 |
|  | *C. sanguineus* | 2 | 7.36 | **<0.001** |
|  | *B. sessilis* | 1 | 0.45 | 0.50 |
|  | *H. laurina* | 1 | 1.63 | 0.20 |

**Table S6** Seedling emergence percentage, survival percentage, shoot growth and time to reach 50% of the maximum emergence (T_50_) for non-pelleted seeds, pellets, and pellets with inoculum of beneficial organisms in six species.

| **Species** | **Treatment** | **Seedling emergence %**  **(Mean ± SE)** | **T_50_**  **(Days)** | **Seedling survival %**  **(Mean ± SE)** | **Seedling shoot growth mm**  **(Mean ± SE)** |
| --- | --- | --- | --- | --- | --- |
| *Acacia acuminata* | Non-pelleted | 81.0 ± 3.49 | 19.91 | 36.9 ± 4.32 | 18.8 ± 0.37 |
|  | Pellets | 45.1 ± 4.88 | 29.29 | 14.4 ± 3.07 | 16.6 ± 0.78 |
|  | Pellets + Additive | 41.7 ± 5.19 | 29.44 | 10.1 ± 2.95 | 16.6 ± 0.82 |
| *Acacia saligna* | Non-pelleted | 79.9 ± 4.28 | 15.79 | 24.0 ± 3.92 | 18.4 ± 0.60 |
|  | Pellets | 61.8 ± 7.82 | 26.46 | 8.5 ± 2.05 | 19.6 ± 1.28 |
|  | Pellets + Additive | 63.2 ± 6.92 | 26.32 | 9.6 ± 2.17 | 18.5 ± 1.26 |
| *Calothamnus sanguinus* | Non-pelleted | 74.5 ± 4.70 | 23.04 | 61.5 ± 3.49 | 19.9 ± 0.37 |
|  | Pellets | 58.6 ± 6.80 | 28.25 | 45.8 ± 3.28 | 19.1 ± 0.49 |
|  | Pellets + Additive | 56.5 ± 6.65 | 28.76 | 44.5 ± 3.91 | 17.7 ± 0.40 |
| *Melaleuca seriata* | Non-pelleted | 62.0 ± 4.88 | 20.87 | - | - |
|  | Pellets | 57.4 ± 4.95 | 23.49 | - | - |
|  | Pellets + Additive | 56.5 ± 4.34 | 24.02 | - | - |
| *Banksia sessilis* | Non-pelleted | 29.6 ± 2.31 | 45.34 | 36.4 ± 7.45 | 43.5 ± 2.09 |
|  | Pellets | 16.0 ± 1.97 | 51.66 | 32.6 ± 6.97 | 46.2 ± 2.58 |
| *Hakea laurina* | Non-pelleted | 95.6 ± 1.28 | 37.80 | 84.5 ± 2.82 | 59.2 ± 0.90 |
|  | Pellets | 81.3 ± 6.08 | 37.00 | 81.9 ± 3.97 | 57.9 ± 0.95 |

**Table S7** Results from generalised linear mixed model and linear mixed model ANOVA analysis for the effect reserve on seedling emergence, survival, and shoot growth of six species.

| **Response** | **Species** | **Df** | **Chi sq / F value** | **P value** |
| --- | --- | --- | --- | --- |
| Emergence | *A. acuminata* | 2 | 93.15 | **<0.001** |
|  | *A. saligna* | 2 | 213.52 | **<0.001** |
|  | *C. sanguineus* | 2 | 96.96 | **<0.001** |
|  | *M. seriata* | 2 | 8.53 | **0.01** |
|  | *B. sessilis* | 2 | 0.12 | 0.94 |
|  | *H. laurina* | 2 | 25.79 | **<0.001** |
| Survival | *A. acuminata* | 2 | 0.93 | 0.40 |
|  | *A. saligna* | 2 | 1.83 | 0.17 |
|  | *C. sanguineus* | 2 | 1.18 | 0.31 |
|  | *B. sessilis* | 2 | 5.05 | **0.01** |
|  | *H. laurina* | 2 | 1.12 | 0.34 |
| Shoot growth | *A. acuminata* | 2 | 18.88 | **<0.001** |
|  | *A. saligna* | 2 | 2.33 | 0.10 |
|  | *C. sanguineus* | 2 | 3.11 | 0.05 |
|  | *B. sessilis* | 2 | 0.18 | 0.83 |
|  | *H. laurina* | 2 | 26.66 | **<0.001** |

**Table S8** P values for the pairwise comparisons of seedling emergence, survival and shoot growth between reserves using estimated marginal means for generalised linear mixed model and linear mixed model in six species.

| **Response** | **Species** | **Comparison** | **Estimate** | **Standard Error** | **t / z ratio** | **p value** |
| --- | --- | --- | --- | --- | --- | --- |
| Emergence | *A. acuminata* | Black Cockatoo – Falls Road | -1.30 | 0.16 | -8.29 | **<0.001** |
|  |  | Black Cockatoo – Quail Street | -1.35 | 0.16 | -8.57 | **<0.001** |
|  |  | Falls Road – Quail Street | -0.05 | 0.15 | -0.31 | 0.95 |
|  | *A. saligna* | Black Cockatoo – Falls Road | -2.14 | 0.17 | -12.62 | **<0.001** |
|  |  | Black Cockatoo – Quail Street | -1.93 | 0.16 | -11.83 | **<0.001** |
|  |  | Falls Road – Quail Street | 0.21 | 0.18 | 1.18 | 0.46 |
|  | *C. sanguineus* | Black Cockatoo – Falls Road | -1.61 | 0.17 | -9.76 | **<0.001** |
|  |  | Black Cockatoo – Quail Street | 0.47 | 0.15 | -3.18 | **<0.01** |
|  |  | Falls Road – Quail Street | 1.14 | 0.16 | 6.95 | **<0.001** |
|  | *M. seriata* | Black Cockatoo – Falls Road | -0.34 | 0.14 | -2.39 | **0.04** |
|  |  | Black Cockatoo – Quail Street | 0.04 | 0.14 | 0.28 | 0.96 |
|  |  | Falls Road – Quail Street | 0.38 | 0.14 | 2.67 | **0.02** |
|  | *B. sessilis* | Black Cockatoo – Falls Road | 0.06 | 0.20 | 0.30 | 0.95 |
|  |  | Black Cockatoo – Quail Street | 0.06 | 0.20 | 0.30 | 0.95 |
|  |  | Falls Road – Quail Street | 0.00 | 0.20 | 0.00 | 1.00 |
|  | *H. laurina* | Black Cockatoo – Falls Road | -1.68 | 0.37 | -4.52 | **<0.001** |
|  |  | Black Cockatoo – Quail Street | 0.15 | 0.25 | 0.62 | 0.81 |
|  |  | Falls Road – Quail Street | 1.84 | 0.37 | 4.97 | **<0.001** |
| Survival | *A. acuminata* | Black Cockatoo – Falls Road | -6.29 | 4.62 | -1.36 | 0.36 |
|  |  | Black Cockatoo – Quail Street | -3.42 | 4.62 | -0.74 | 0.74 |
|  |  | Falls Road – Quail Street | 2.87 | 4.62 | 0.62 | 0.81 |
|  | *A. saligna* | Black Cockatoo – Falls Road | 1.43 | 3.74 | 0.38 | 0.92 |
|  |  | Black Cockatoo – Quail Street | 6.78 | 3.74 | 1.81 | 0.18 |
|  |  | Falls Road – Quail Street | 5.34 | 3.74 | 1.43 | 0.33 |
|  | *C. sanguineus* | Black Cockatoo – Falls Road | -6.52 | 5.03 | -1.29 | 0.41 |
|  |  | Black Cockatoo – Quail Street | -6.84 | 5.03 | -1.36 | 0.37 |
|  |  | Falls Road – Quail Street | 0.33 | 5.03 | -0.07 | 0.99 |
|  | *B. sessilis* | Black Cockatoo – Falls Road | -1.76 | 10.7 | -0.17 | 0.98 |
|  |  | Black Cockatoo – Quail Street | 28.47 | 10.7 | 2.67 | **0.03** |
|  |  | Falls Road – Quail Street | 30.23 | 10.7 | 2.83 | **0.02** |
|  | *H. laurina* | Black Cockatoo – Falls Road | 5.76 | 5.13 | 1.12 | 0.51 |
|  |  | Black Cockatoo – Quail Street | 7.28 | 5.13 | 1.42 | 0.35 |
|  |  | Falls Road – Quail Street | 1.53 | 5.13 | 0.29 | 0.95 |

**Table S8** continued.

| **Response** | **Species** | **Comparison** | **Estimate** | **Standard Error** | **t / z ratio** | **p value** |
| --- | --- | --- | --- | --- | --- | --- |
| Shoot growth | *A. acuminata* | Black Cockatoo – Falls Road | -4.50 | 0.75 | -6.04 | **<0.001** |
|  |  | Black Cockatoo – Quail Street | -3.01 | 0.82 | -3.68 | **<0.001** |
|  |  | Falls Road – Quail Street | 1.49 | 0.68 | 2.19 | 0.07 |
|  | *A. saligna* | Black Cockatoo – Falls Road | -1.34 | 1.27 | -1.06 | 0.54 |
|  |  | Black Cockatoo – Quail Street | 1.35 | 1.40 | 0.97 | 0.59 |
|  |  | Falls Road – Quail Street | 2.69 | 1.27 | 2.11 | 0.09 |
|  | *C. sanguineus* | Black Cockatoo – Falls Road | 0.08 | 0.61 | 0.14 | 0.99 |
|  |  | Black Cockatoo – Quail Street | 1.33 | 0.65 | 2.03 | 0.11 |
|  |  | Falls Road – Quail Street | 1.24 | 0.55 | 2.25 | 0.06 |
|  | *B. sessilis* | Black Cockatoo – Falls Road | 0.27 | 3.86 | 0.07 | 0.99 |
|  |  | Black Cockatoo – Quail Street | 2.77 | 5.02 | 0.55 | 0.84 |
|  |  | Falls Road – Quail Street | 2.49 | 4.92 | 0.51 | 0.86 |
|  | *H. laurina* | Black Cockatoo – Falls Road | -2.11 | 1.51 | -1.39 | 0.35 |
|  |  | Black Cockatoo – Quail Street | 8.64 | 1.56 | 5.55 | **<0.001** |
|  |  | Falls Road – Quail Street | 10.74 | 1.55 | 6.93 | **<0.001** |

**Table S9** Estimated regression parameters, standard errors and z-values or t-values for the generalised linear mixed model fit for seedling emergence, and linear mixed models fit for seedling survival and shoot growth of non-pelleted seeds, pellets, and pellets with inoculum of beneficial organisms in six species.

| **Response** | **Species** |  | **Estimate** | **Standard Error** | **z value / t value** | **P value** |
| --- | --- | --- | --- | --- | --- | --- |
| Emergence | *A. acuminata* | Non-pelleted (Intercept) | 0.67 | 0.169 | 3.97 | **<0.001** |
|  |  | Pellet | -1.78 | 0.166 | -10.76 | **<0.001** |
|  |  | Pellet + Additive | -1.94 | 0.167 | -11.63 | **<0.001** |
|  |  | Falls Road Reserve | 1.30 | 0.157 | 8.29 | **<0.001** |
|  |  | Quail Street Reserve | 1.35 | 0.157 | 8.57 | **<0.001** |
|  | *A. saligna* | Non-pelleted (Intercept) | 0.30 | 0.161 | 1.89 | 0.05 |
|  |  | Pellet | -1.11 | 0.175 | -6.35 | **<0.001** |
|  |  | Pellet + Additive | -1.02 | 0.175 | -5.84 | **<0.001** |
|  |  | Falls Road Reserve | 2.14 | 0.170 | 12.62 | **<0.001** |
|  |  | Quail Street Reserve | 1.93 | 0.163 | 11.83 | **<0.001** |
|  | *C. sanguineus* | Non-pelleted (Intercept) | 0.60 | 0.295 | 2.02 | **0.04** |
|  |  | Pellet | -0.95 | 0.159 | -5.94 | **<0.001** |
|  |  | Pellet + Additive | -0.86 | 0.159 | -5.37 | **<0.001** |
|  |  | Falls Road Reserve | 1.61 | 0.165 | 9.76 | **<0.001** |
|  |  | Quail Street Reserve | 0.47 | 0.146 | 3.18 | **0.001** |
|  | *M. seriata* | Non-pelleted (Intercept) | 0.40 | 0.189 | 2.10 | **0.03** |
|  |  | Pellet | -0.19 | 0.141 | -1.34 | 0.18 |
|  |  | Pellet + Additive | -0.23 | 0.141 | -1.62 | 0.11 |
|  |  | Falls Road Reserve | 0.34 | 0.142 | 2.39 | **0.01** |
|  |  | Quail Street Reserve | -0.04 | 0.139 | -0.28 | 0.78 |
|  | *B. sessilis* | Non-pelleted (Intercept) | -0.81 | 0.156 | -5.23 | **<0.001** |
|  |  | Pellet | -0.81 | 0.168 | -4.79 | **<0.001** |
|  |  | Falls Road Reserve | -0.06 | 0.201 | -0.30 | 0.76 |
|  |  | Quail Street Reserve | -0.06 | 0.201 | -0.30 | 0.76 |
|  | *H. laurina* | Non-pelleted (Intercept) | 3.02 | 0.396 | 7.62 | **<0.001** |
|  |  | Pellet | -1.72 | 0.273 | -6.31 | **<0.001** |
|  |  | Falls Road Reserve | 1.68 | 0.372 | 4.52 | **<0.001** |
|  |  | Quail Street Reserve | -0.15 | 0.246 | -0.62 | 0.53 |
| Survival | *A. acuminata* | Non-pelleted (Intercept) | 33.65 | 4.83 | 6.97 | **<0.001** |
|  |  | Pellet | -22.54 | 4.62 | -4.88 | **<0.001** |
|  |  | Pellet + Additive | -26.81 | 4.62 | -5.80 | **<0.001** |
|  |  | Falls Road Reserve | 6.29 | 4.62 | 1.36 | 0.18 |
|  |  | Quail Street Reserve | 3.42 | 4.62 | 0.74 | 0.46 |

| **Response** | **Species** |  | **Estimate** | **Standard Error** | **z value / t value** | **P value** |
| --- | --- | --- | --- | --- | --- | --- |
| Survival | *A. saligna* | Non-pelleted (Intercept) | 26.75 | 3.83 | 6.99 | **<0.001** |
|  |  | Pellet | -15.55 | 3.74 | -4.16 | **<0.001** |
|  |  | Pellet + Additive | -14.46 | 3.74 | -3.87 | **<0.001** |
|  |  | Falls Road Reserve | -1.43 | 3.74 | -0.38 | 0.70 |
|  |  | Quail Street Reserve | -6.77 | 3.74 | -1.81 | 0.08 |
|  | *C. sanguineus* | Non-pelleted (Intercept) | 57.07 | 4.59 | 12.42 | **<0.001** |
|  |  | Pellet | -15.77 | 5.03 | -3.14 | **<0.01** |
|  |  | Pellet + Additive | -17.05 | 5.03 | -3.39 | **<0.01** |
|  |  | Falls Road Reserve | 6.52 | 5.03 | 1.29 | 0.20 |
|  |  | Quail Street Reserve | 6.84 | 5.03 | 1.36 | 0.18 |
|  | *B. sessilis* | Non-pelleted (Intercept) | 45.29 | 9.63 | 4.70 | **<0.01** |
|  |  | Pellet | -3.76 | 8.72 | -0.43 | 0.67 |
|  |  | Falls Road Reserve | 1.77 | 10.68 | 0.17 | 0.87 |
|  |  | Quail Street Reserve | -28.47 | 10.68 | -2.67 | **0.01** |
|  | *H. laurina* | Non-pelleted (Intercept) | 88.82 | 5.27 | 16.84 | **<0.001** |
|  |  | Pellet | -2.57 | 4.19 | 0.61 | 0.54 |
|  |  | Falls Road Reserve | -5.76 | 5.13 | -1.12 | 0.27 |
|  |  | Quail Street Reserve | -7.28 | 5.13 | -1.42 | 0.17 |
| Shoot growth | *A. acuminata* | Non-pelleted (Intercept) | 16.07 | 0.59 | 27.19 | **<0.001** |
|  |  | Pellet | -2.69 | 0.84 | -3.23 | **<0.01** |
|  |  | Pellet + Additive | -3.03 | 0.90 | -3.35 | **<0.001** |
|  |  | Falls Road Reserve | 4.50 | 0.73 | 6.15 | **<0.001** |
|  |  | Quail Street Reserve | 3.1 | 0.79 | 3.79 | **<0.001** |
|  | *A. saligna* | Non-pelleted (Intercept) | 18.32 | 0.99 | 18.34 | **<0.001** |
|  |  | Pellet | 0.92 | 1.35 | 0.68 | 0.49 |
|  |  | Pellet + Additive | -0.37 | 1.32 | -0.28 | 0.78 |
|  |  | Falls Road Reserve | 1.34 | 1.24 | 1.08 | 0.28 |
|  |  | Quail Street Reserve | -1.35 | 1.38 | -0.98 | 0.33 |
|  | *C. sanguineus* | Non-pelleted (Intercept) | 20.21 | 0.79 | 25.31 | **<0.001** |
|  |  | Pellet + Additive | -2.17 | 0.57 | -3.81 | 0.07 |
|  |  | Pellet | -1.04 | 0.57 | -1.83 | **<0.001** |
|  |  | Falls Road Reserve | -0.08 | 0.61 | -0.14 | 0.89 |
|  |  | Quail Street Reserve | -1.33 | 0.65 | -2.04 | **0.04** |

**Table S9 c**ontinued

| **Response** | **Species** |  | **Estimate** | **Standard Error** | **z value / t value** | **P value** |
| --- | --- | --- | --- | --- | --- | --- |
| Shoot growth | *B. sessilis* | Non-pelleted (Intercept) | 44.01 | 3.09 | 14.19 | **<0.001** |
|  |  | Pellet | 2.48 | 3.71 | 0.66 | 0.50 |
|  |  | Falls Road Reserve | -0.27 | 3.74 | -0.07 | 0.94 |
|  |  | Quail Street Reserve | -2.76 | 4.79 | -0.58 | 0.57 |
|  | *H. laurina* | Non-pelleted (Intercept) | 61.18 | 1.87 | 32.68 | **<0.001** |
|  |  | Pellet | -1.59 | 1.25 | -1.28 | 0.20 |
|  |  | Falls Road Reserve | 2.11 | 1.51 | 1.39 | 0.16 |
|  |  | Quail Street Reserve | -8.64 | 1.55 | -5.56 | **<0.001** |

**Table S9 c**ontinued

**References**

Abarenkov K, Zirk A, Piirmann T, Pöhönen R, Ivanov F, Nilsson RH, Kõljalg U (2022) Full UNITE+INSD dataset for Fungi

Chen YL, Dell B, Malajczuk N (2006) Effect of *Scleroderma* spore density and age on mycorrhiza formation and growth of containerized *Eucalyptus globulus* and *E. urophylla* seedlings. *New Forests* 31:453-467

Deaker R, Hartley E, Gemell G, Herridge DF, Karanja N (2016) Inoculant production and quality control. Page 167-186 In*:* Howieson JG, Dilworth MJ (eds) Working with rhizobia*.* Australian Centre for International Agricultural Research, Canberra, Australia

Hungria M, O’Hara GW, Zilli JE, Araujo RS, Deaker R, Howieson JG (2016) Isolation and growth of rhizobia. Page 39-60 In*:* Howieson JG, Dilworth MJ (eds) Working with rhizobia*.* Australian Centre for International Agricultural Research, Canberra, Australia

Ihrmark K, Bödeker ITM, Cruz-Martinez K, Friberg H, Kubartova A, Schenck J, Strid Y, Stenlid J, et al. (2012) New primers to amplify the fungal ITS2 region – evaluation by 454-sequencing of artificial and natural communities. *Microbiology Ecology,* 82: 666-677

Miles AA, Misra SS, Irwin JO (1938) The estimation of the bactericidal power of the blood. *Epidemiology and Infection* 38:732-749

Yates RJ, Howieson JG, Nandasena KG, O'Hara GW (2004) Root-nodule bacteria from indigenous legumes in the north-west of Western Australia and their interaction with exotic legumes. *Soil Biology and Biochemistry* 36:1319-1329
